# Cardiac surgical simulation program during general surgery residency increases resident physician exposure to cardiac surgery and technical expertise

**DOI:** 10.1016/j.xjon.2022.01.002

**Published:** 2022-01-22

**Authors:** Andrew P. Rabenstein, Alisa Khomutova, A. Laurie W. Shroyer, Richard Scriven, Allison McLarty, Henry Tannous, Jorge M. Balaguer

**Affiliations:** a Department of Thoracic and Cardiovascular Surgery, Allegheny General Hospital, AHN Cardiovascular Institute, Pittsburgh, Pa; b Department of Surgery, General Surgery Residency Program, Stony Brook University Hospital, Stony Brook, NY; c Division of Cardiothoracic Surgery, Department of Surgery, Stony Brook University Hospital, Stony Brook, NY

**Keywords:** general education, educational research, residency education, simulation, I-6, integrated program, PA, physician assistant, PGY, postgraduate year, TAVR, transcatheter aortic valve replacement, VAD, ventricular assist device

## Abstract

**Objective:**

The changing surgical education landscape in surgical training pathways greatly diminished cardiac surgical knowledge, interest, and skills among general surgery trainees. To address this issue, our department developed a cardiac surgery simulation program.

**Methods:**

All simulation sessions lasted at least 2 hours and occurred during resident physician protected education time. Participants were postgraduate year 2 through 5 general surgery residents assisted by staff and led by cardiac surgery faculty. Five of the 6 sessions were porcine heart wet labs simulating coronary anastomoses, surgical aortic valve replacement, mitral valve repair and replacement, and left ventricular assist device implantation. The transcatheter aortic valve replacement session was designed as a video simulation and a manikin for wire manipulation and implantation. At the end of each lab, all participants were surveyed about their experiences.

**Results:**

An average of 10 resident physicians participated in each session (range, 8-13), for a total of 120 simulation hours. One hundred percent of residents surveyed agreed that the labs improved knowledge and understanding of the disease process, improved understanding of cardiac surgical principles, and helped acquire skills for surgical residency and treatment. Factors that residents cited for increased attendance rate included protected education time, hands-on experience, and a high faculty-to-resident ratio.

**Conclusions:**

This program successfully demonstrates that cardiac surgery training and simulation can be integrated into general surgery residency programs, despite the lack of cardiac surgery requirements. Additional metrics for future study includes technical grades on resident physicians’ performance to further assess the value of this program.

## Graphical abstract

Graphical abstract depicting the methods, results, and implications of the study. *CABG*, Coronary artery bypass grafting; *AVR*, aortic valve replacement; *MVR*, mitral valve replacement; *LVAD*, left ventricular assist device; *OR*, operating room; *PA*, physician assistant; *RN*, registered nurse.

**Figure:**
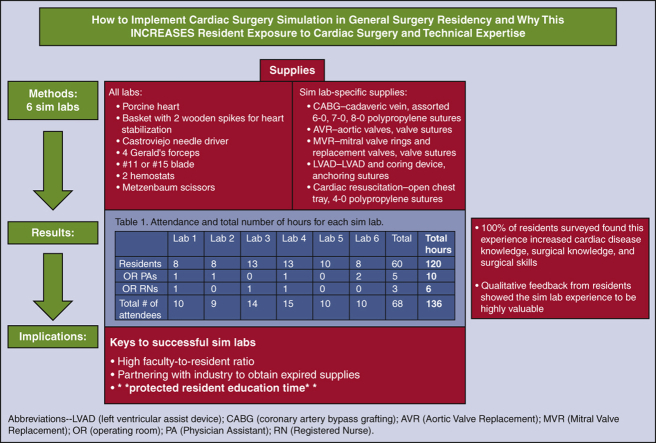

**Figure:**
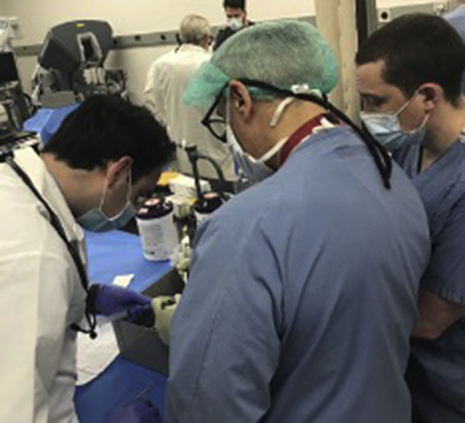
Example of simulation session with 2:1 resident-to-faculty ratio.

**Central Message**

Cardiac surgery simulation can be integrated into general surgery residency programs. This is important to drive general surgery resident interest in pursuing a cardiac surgery career.

**Perspective**

The past 2 decades have seen a gradual realignment in cardiac surgery education, from traditional fellowships to integrated 6-year programs and 4 + 3 programs. During this same time simulation has emerged as a great route of exposure and education. This project successfully integrated cardiac surgery simulation into general surgery training, improving exposure and technical skills of trainees.

See Commentaries on pages 185 and 187.

Traditionally, cardiothoracic surgeons completed a general surgery residency, followed by 2 to 3 years of fellowship, for a total of 5 to 7 years of total graduate surgical training. Some of these programs were subspecialty focused—cardiac or thoracic tract-based. The first integrated program (I-6) was pioneered in 2007 by Stanford University.^1^^,^^2^ After graduating from medical school, physicians could access training in cardiothoracic surgery without completing a general surgery training program. Although I-6 programs provided an average of 12 to 36 months of general surgery exposure to the junior residents, these resident physicians would not meet the criteria for dual board certification in general and thoracic surgery.^1^ During the past few years, a newer 4 + 3 type of hybrid program has also emerged and is currently offered by 11 programs across the United States.^1^ In these 7-year programs, trainees apply for thoracic fellowship during their general surgery residency, subsequently complete 4 years in general surgery and 3 years in cardiothoracic surgery—and are dual certified at the end of the training.

Based on National Resident Matching Program archived data between 2008 and 2018, the number of available general surgery resident positions increased from 1069 to 1432, roughly 2% each year. From 2008 to 2018, the number of US senior medical student applicants into general surgery increased by 6.1% During this same period, the number of US allopathic graduates who applied into cardiothoracic surgery fellowships increased by 22%, from 67 to 82. During this same period, the number of available traditional cardiothoracic surgery residency positions decreased by 27% (from 130 to 95). As a result, the number of unfilled positions in the specialty steadily declined each year,^3^^,^^4^ driven more by a decrease in the number of available spots than a decrease in the number of applicants.^4^ Furthermore, much of the dramatic increase in the number of applicants to integrated thoracic residency programs in the United States has been driven by international graduates. Most of them have had several years of general surgery and cardiac surgery experience.^5^ Essentially, interest and pursuit of cardiothoracic surgery has not grown proportionally with interest in general surgery and other subspecialties. In fact, models have predicted a shortage of cardiothoracic surgeons and a growing need. It is projected that by 2025 the demand for cardiothoracic surgeons could increase by 46%, with each surgeon performing 121% more cases by 2035.^6^ Some of the declining interest in the specialty was due to an excess of surgeons leading to an increase in unfilled training spots, and a subsequent closure of some programs. This decrease in spots was coupled by a perception of the continued rise in interventional cardiology and vascular surgery, and these fields’ impingement into the cardiac surgery scope of care.

The case requirements for graduating general surgery resident physicians have evolved over time. Currently, the American Board of Surgery does not require cardiac cases and only 20 general thoracic surgery cases.^7^ Whereas the total thoracic case numbers among general surgery residents have not changed significantly, resident physician participation as a first assistant in key thoracic cases has decreased over the past 11 years. Conversely, participation in video-assisted thoracoscopic surgery and minor cases has increased. This could be due to a lack of resident interest or perceived insufficient technical skills due to decreasing exposure.^8^ In this context, it is essential to find alternatives to increase or maintain general surgery resident physicians’ exposure to cardiothoracic surgery aside from the early exposure in medical school.

Simulation training provides an immersive experience in a comfortable, controlled environment for surgical trainees at any level without compromising patient safety. National societies organize boot-camps and cadaveric or inanimate model-based training courses; however, there is a lack of adoption of cardiac simulation training and operative exposure in general surgery programs across the country. Kaban and colleagues^9^ demonstrated that simulation training can even influence what subspecialty a general surgery resident physician pursues. Recognizing the importance of simulation the Association of Program Directors of Surgery in conjunction with the American College of Surgeons created a 41-part simulation lab series for general surgery trainees—unfortunately none of them involved cardiac surgery. This series has been shown to improve qualitative and quantitative operative performance, situational awareness, familiarity, and confidence in real operating rooms.^10^^,^^11^ Therefore, one could argue that the benefits of completing isolated, focused cardiac surgical simulated exercises such as coronary anastomoses are to provide trainees with deliberate individually tailored tasks in low-risk, low-pressure circumstances to improve the trainees’ comfort level and technical skills. Nesbitt and colleagues^12^ successfully demonstrated this in a coronary anastomosis simulation experience by fourth-year medical students. Eventually, these skills, including hand-eye coordination, dexterity in handling very delicate sutures, and Castroviejo needle holders are naturally integrated. Should a participant plan on entering a noncardiac surgical specialty, the skills and techniques acquired can still be valuable and applied–from pancreaticojejunal anastomoses in Whipple procedures to arteriovenous fistula creations in vascular surgery.

## Materials and Methods

### Procurement

**Figure 1:**
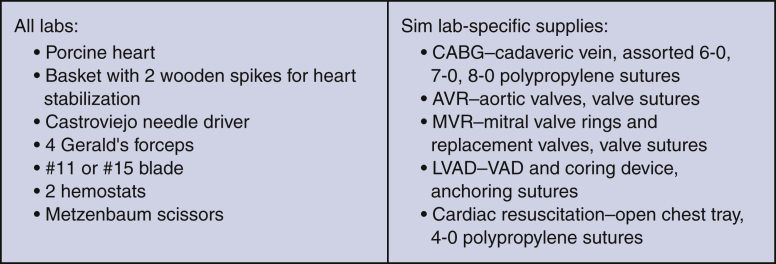
List of supplies needed for each simulation lab (Sim lab) (per 2-person group). *CABG*, Coronary artery bypass grafting; *AVR*, aortic valve replacement; *MVR*, mitral valve replacement; *LVAD*, left ventricular assist device.

**Figure 2:**
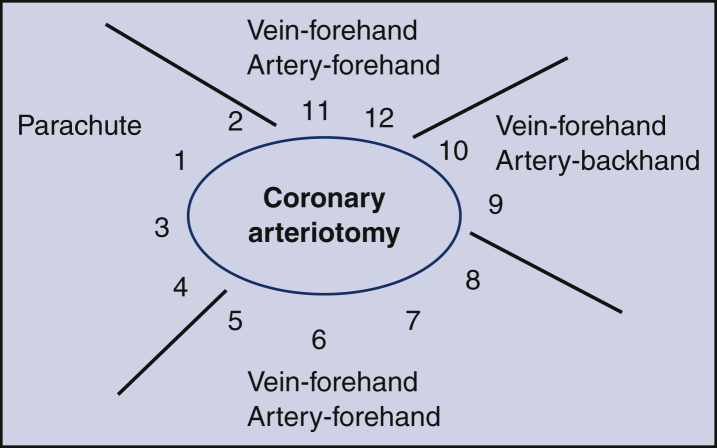
Schematic diagram for coronary anastomosis.

No direct monetary funding was provided for these simulation labs. Six porcine hearts were donated from industry for each simulation session, as well as some surgical instruments, drapes, sponges, and lap pads. Expired prostheses and grafts were donated by the affiliated company (CryoLife, Abbott, Medtronic, and Edwards Life Sciences) depending on the applicable lab activity (eg, mitral valve, cadaveric vein, or ventricular assist device sewing cuffs). The majority of instruments used were obtained from the Stony Brook skills lab for simulation activities. The suture materials used were predominantly expired, and some were donated by industry. The materials used for each simulation are detailed in Figure 1, and an example of a schematic diagram for a coronary anastomosis as used in the lab is outlined (Figure 2).

### Experience

**Figure 3:**
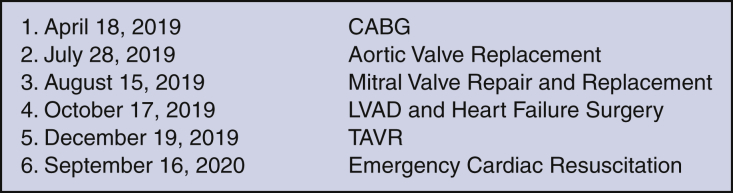
Cardiac surgery simulation schedule. *CABG*, Coronary artery bypass grafting; *LVAD*, left ventricular assist device; *TAVR*, transcatheter aortic valve replacement.

The cardiac simulation lab series consisted of 6 labs (5 porcine labs and 1 computer simulation model) spaced approximately 2 months apart over 1.5 years (gap in sessions due to COVID-19 pandemic) (Figure 3). All sessions were coordinated by the same senior cardiac surgery faculty member, who had administered prior similar sessions. All labs were scheduled during protected resident physician education time to avoid clinical conflicts. The sessions were mandatory, unless the resident physician had previously approved obligations or was engaged in patient care. Trainees in postgraduate year (PGY) 2 through 5 attended to maximize the education based on level-appropriate technical skills required for the sessions. Each session lasted 2 to 3 hours. The sessions were led by the main subspecialist cardiac surgeon in a given field; a minimum of 3 to 5 attending and emeritus cardiac surgeons were also present to serve as instructors. The tasks of each session were chosen by cardiothoracic department faculty and general surgery resident education faculty in an attempt to expose resident physicians to the most common techniques and procedures in cardiac surgery.

Additional clinical support staff was invited to supplement the simulation activity and create an even more realistic environment. Operating room physician assistants (PAs) and scrub nurses were present to assist the resident physicians. Medical students on service were invited and available at some stations to assist. All personnel were situated on either side of the heart to simulate real positioning.

### Data Analysis

At the end of each simulation, all participants were given a 3-item questionnaire asking whether or not they believed the activity improved their knowledge and understanding of the disease process; improved their understanding of cardiac surgical principles; and helped acquire skills for surgical residency and treatment. All of the data were subsequently analyzed using Microsoft Excel.

## Results

### Simulation Lab Session 1: Coronary Artery Bypass Grafting

This session focused on distal anastomoses and lasted 2 hours. It was attended by 8 resident physicians, 1 operating room PA, and 1 scrub nurse. The session began with didactics on how to perform coronary anastomoses (Figure 2). Continuous 7–0 polypropylene sutures were used for all distal anastomosis in an end-to-side fashion between the cryopreserved cadaver vein and left anterior descending in a porcine model. Anastomosis was tested with pressurized saline to check for leaks. Techniques to repairs anastomotic leaks were also taught, and the anastomosis was then dissected to assess anatomic quality.

### Simulation Lab Session 2: Aortic Valve Replacement

This session focused on open aortic valve replacement and lasted 2 hours. Eight residents and 1 operating room PA attended it. Didactics, at the beginning of the session focused on aortic valve anatomy, physiology, and technical considerations. The simulation consisted of teams of 2 resident physicians to excise the original valve and implant an aortic valve prosthesis. Aortotomy, native valve removal, placement of sutures (2-0 Ethibond pledgeted sutures) through the aortic valve annulus, seating the prosthesis and tying the sutures as well as the closure of the aortotomy were performed by the resident physicians during this session.

### Simulation Lab Session 3: Mitral Valve Repair and Replacement

This session focused on open mitral valve repair and replacement and lasted 2 hours. Thirteen resident physicians and 1 scrub nurse attended it. Didactics at the beginning of the session focused on mitral valve and leaflet anatomy, physiology, various repair techniques, and indications for mitral valve repair versus replacement. The residents also practiced implantation of annuloplasty rings and valve replacement with a bioprosthesis. Valve analysis to determine the feasibility of repair was discussed. All resident physicians were divided into pairs. Each pair was then given the opportunity to perform a repair with ring annuloplasty or a valve replacement.

### Simulation Lab Session 4: Ventricular Assist Devices

This session focused on ventricular assist devices (VADs). It was attended by 13 resident physicians, 1 operating room PA, and 1 scrub nurse. This session began with a brief review of heart failure physiology and operative interventions, focusing on VADs and extra-corporeal membrane oxygenation implantation (we avoided discussing the mechanics of how to manage these devices because our resident physicians already experienced this in the cardiothoracic surgery intensive care unit). The manufacturer provided expired VAD sewing cuffs. Resident physicians worked in teams of 2 to core the left ventricle and suture the sewing cuff to the cored apex of the left ventricle. Hands-on experience with the remainder of the VAD components was provided with company support staff. Demonstration devices were brought to the session for educational purposes.

### Simulation Lab Session 5: Transcatheter Aortic Valve Replacement

This session focused on transcatheter aortic valve replacement (TAVR) operative planning and implantation techniques and lasted 2 hours. Ten resident physicians and 5 nurse practitioners attended it. Unlike the rest of the sessions, the TAVR program was similar to the training attending surgeons and cardiologists undergo. The first component was a 15-minute lecture regarding the value of cardiothoracic angiography in the planning of a TAVR procedure. Images were reviewed and focused on the diameter and circumference of the aortic valve annulus, the height of the ostium of the left main and right coronary arteries, the size of the sinus of Valsalva, and the diameter of the sinotubular junction. Access sites for large access sheaths (eg, femoral, axillary, aortic, or transapical) were discussed. The iliofemoral vascular systems were analyzed in detail, including minimal diameters of the femoral and iliac vessels. Atherosclerosis, tortuosity, calcification in the iliofemoral system, the abdominal, descending aorta, aortic arch and ascending aorta, aortic valve, and left ventricular outflow tract was reviewed. The equipment required for TAVR was distributed among the participants, and specific instrumentation maneuvers were practiced (eg, deploying the valve and adjusting the position of the balloon inside the prosthesis). Regarding the procedure itself, a computer-based simulator reproduced catheter manipulation and its effect on a simulated active real-time fluoroscopy screen (like in the catheterization laboratory). Resident physicians practiced the advancement of guide wires across the aortic valve, advancing the prosthesis, adjusting the position using established criteria, and deploying the prosthetic valve itself. Complication case scenarios (eg, low or high valve deployment and coronary artery obstruction) were also included in this session.

### Simulation Lab Session 6: Emergency Cardiac Resuscitation

The final session focused on addressing bedside operative issues in cardiac surgery—bedside reopening of a chest, internal cardiac massage, and repair of cardiac injuries. Eight residents and 2 PAs attended it. Resident physicians were presented with a scenario about what to do if a postcardiotomy patient goes into cardiac arrest. Resident physicians were familiarized with the open chest tray and how to reopen a chest, including cutting sternal wires and placement of alligator pacing wires. Resident physicians were then given opportunities to practice internal cardiac massage and open chest defibrillation. Finally, resident physicians practiced the repair of wounds in the heart.

### Assessment and Feedback

Direct feedback included comments such as “Truly an amazing experience,” “Fantastic lab-great tissue to work with,” “Good to have multiple attendings/staff to teach,” “Great learning experience,” “Good one-on-one instruction,” and “One of the most, if not the best, educational sessions in the residency.” More specific feedback included, “Definitely helped me in developing my techniques for valve repair/replacement,” “Very informative regarding coronary artery bypass grafting techniques,” and “Open chest resuscitation was excellent and important.” Regarding the TAVR session, comments included: “Great presentation and live demonstration,” “Really clarified steps preop, intraop, and postop,” and “Good hands-on experience and explanation of TAVR planning.”

Surveys among all residents showed that 100% of participants agreed that the labs improved knowledge and understanding of the disease process, improved the understanding of cardiac surgical principles, and helped acquire skills for surgical residency and treatment. Factors that residents cited for increased attendance rate included labs being performed during education protected time, hands-on experience, and a high faculty-to-resident ratio. Surgical PAs and operating room nurses stated that the program helped them improve their skills, teaching abilities, and teamwork. At least 1 resident physician who participated in the program cemented a desire to pursue cardiac surgery fellowship, and 2 others gained mentors in the field and became involved in the cardiothoracic research department.

## Discussion

The past 15 years have seen a crossroads in cardiac surgery education in general surgery residency. On 1 hand there has been a decreasing interest in cardiac surgery education among general surgery resident physicians due to self-selection of interested parties into I-6 programs and the rise of these I-6 programs. Concurrently, general surgery residency programs often forgo any cardiac surgery rotations, although the exact number of general surgery resident physicians who are exposed to cardiac surgery is unknown. The decreasing exposure to cardiac surgery and isolation of cardiac surgery training positions from the traditional general surgery pathway has contributed to a substantially decreased number of applicants and general interest in cardiac surgical principles among general surgery residencies.

We developed our cardiac surgery simulation series to combat these issues. A full 100% of resident physicians had positive opinions on their experience in this lab and a total of 120 resident hours were spent on these simulation sessions (Table 1). All resident physicians found these simulations to be useful to their didactic education as well as their physical skills in both cardiac surgery and noncardiac surgery. Qualitative feedback from resident physicians demonstrated that key factors for high-quality simulation labs were a high faculty-to-resident ratio and performing simulation labs during protected resident education time where they were free of clinical duties. With simulation activities being built into regular general surgery resident physician education, this provides an opportunity for cardiac surgery to enter the shuffle of surgical specialty simulation activities.

**Figure 4:**
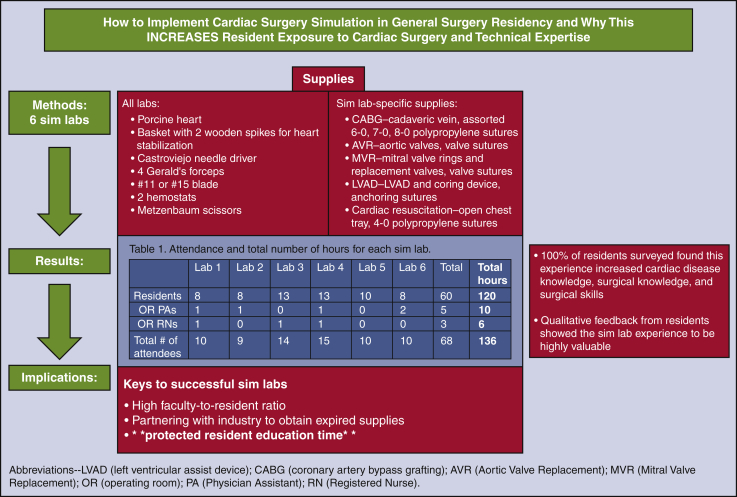
Depiction of the methods, results, and implications of the study. *CABG*, Coronary artery bypass grafting; *AVR*, aortic valve replacement; *MVR*, mitral valve replacement; *LVAD*, left ventricular assist device; *OR*, operating room; *PA*, physician assistant; *RN*, registered nurse.

Our residency program remains among the few where general surgery resident physicians take care of cardiac surgery patients and have the opportunity to participate in cardiac operations. Unfortunately, the percentage of general surgery residencies that offer this is currently unknown. By enabling resident physicians to be exposed to simulation, our hope is that residents will start their cardiac rotation more prepared and have the opportunity to participate more in parts of these complex operations. All of our resident physicians responded positively to the simulation labs and subjectively reported improved techniques in real-time operations. We plan to further develop the cardiac simulation lab at our program and incorporate objective measures such as attending evaluations of resident progress and skill, as well as keep close track of how many resident physicians pursue cardiac research, mentorship, and further training as a result of this exposure. The influence of the cardiac simulation experience is demonstrated in Figure 4.

Future areas of study might involve the implementation of additional labs, including a dedicated 10-hour coronary boot-camp to prepare resident physicians to suture coronary anastomoses in the operating room, the implementation of standardized grading systems for each lab, such as the coronary artery bypass grafting simulation lab grading system,^13^ and use of these data to assess technical improvement from the beginning to the end of the lab. Additionally, to obtain more objective data regarding performance (beyond survey results), standardized feedback reports are currently in development by the cardiac surgery faculty to score the performance of resident physicians who did and did not participate in the simulation training. Given that resident physicians participated in the training during PGY 2 to 5, it is too early to comment on the pursuit of cardiac surgery in all of our trainees, and too late to comment for those who had already matched in other specialties, but records are being kept of fellowship pursuits. Finally, it may be prudent to include all PGY levels (including PGY 1) in the cardiac simulation lab experience to assess whether or not earlier exposure to a complex field would improve surgical aptitude and dexterity and foster an interest in the cardiothoracic field earlier in training.

**Table 1:** Attendance and total number of hours for each simulation lab (program has ∼6 residents per year)

Participant	Lab 1	Lab 2	Lab 3	Lab 4	Lab 5	Lab 6	Total	Total hours
Resident physicians	8	8	13	13	10	8	60	120
OR PAs	1	1	0	1	0	2	5	10
OR RNs	1	0	1	1	0	0	3	6
Total No. of attendees	10	9	14	15	10	10	68	136

*OR*, Operating room; *PA*, physician assistant; *RN*, registered Nurse.

## Conclusions

Overall, this simulation exercise was well received by general surgery resident physicians and helped to drive increased appreciation and technical skills regarding cardiac surgery among general surgery resident physicians.

### Conflict of Interest Statement

The authors reported no conflicts of interest.

The *Journal* policy requires editors and reviewers to disclose conflicts of interest and to decline handling or reviewing manuscripts for which they may have a conflict of interest. The editors and reviewers of this article have no conflicts of interest.

## References

[1] Zhu Y., Goldstone A.B., Woo Y.J. (2019). Integrated thoracic surgery residency: current status and future evolution. Semin Thorac Cardiovasc Surg.

[2] The Thoracic Surgery Directors Association (2012). CT surgery training pathways. https://tsda.org/the-tsda/ct-residency-programs/ct-surgery-training-pathways/.

[3] National Resident Matching Program Results and data: 2018 main residency match. http://www.nrmp.org/report-archives/.

[4] Bui J., Bennett W.C., Long J., Strassle P.D., Haithcock B. (2021). Recent trends in cardiothoracic surgery training: data from the National Resident Matching Program. J Surg Educ.

[5] Chikwe J., Brewer Z., Goldstone A.B., Adams D.H. (2011). Integrated thoracic residency program applicants: the best and the brightest?. Ann Thorac Surg.

[6] Grover A., Karyn G., Dall T.M., Jonas R., Lytle B., Shemin R. (2009). Shortage of cardiothoracic surgeons is likely by 2020. Circulation.

[7] Accreditation Council for Graduate Medical Education (2019). Defined category minimum numbers for general surgery residents and credit role. https://www.acgme.org/%20Portals/0/DefinedCategoryMinimumNumbersforGeneralSurgeryResidentsandCreditRole.pdf.

[8] Ragalie W.S., Termuhlen P.M., Little A.G. (2016). Changes in thoracic surgery experience during general surgery residency: a review of the case logs from the Accreditation Council for Graduate Medical Education. Ann Thorac Surg.

[9] Kaban J.M., Dayama A., Reddy S.H., Teperman S., Stone M.E. (2016). Can specialized surgical simulation influence resident career choice?. Int J Surg.

[10] Villanueva C., Xiong J., Rajput S. (2020). Simulation-based surgical education in cardiothoracic training. ANZ J Surg.

[11] Spadaro S., Karbing D.S., Fogagnolo A., Ragazzi R., Mojoli F., Astolfi L. (2017). Simulation training for residents focused on mechanical ventilation: a randomized trial using mannequin-based versus computer-based simulation. Simul Healthc.

[12] Nesbitt J.C., St Julien J., Absi T.S., Ahmad R.M., Grogan E.L., Balaguer J.M. (2013). Tissue-based coronary surgery simulation: medical student deliberate practice can achieve equivalency to senior surgery residents. J Thorac Cardiovasc Surg.

[13] Fann J.I., Caffarelli A.D., Georgette G., Howard S.K., Gaba D.M., Youngblood P. (2008). Improvement in coronary anastomosis with cardiac surgery simulation. J Thorac Cardiovasc Surg.

